# The association between reproductive history and abdominal adipose tissue among postmenopausal women: results from the Women’s Health Initiative

**DOI:** 10.1093/humrep/deae118

**Published:** 2024-06-18

**Authors:** Hailey R Banack, Claire E Cook, Sonia M Grandi, Natalie V Scime, Rana Andary, Shawna Follis, Matthew Allison, JoAnn E Manson, Su Yong Jung, Robert A Wild, Leslie V Farland, Aladdin H Shadyab, Jennifer W Bea, Andrew O Odegaard

**Affiliations:** Epidemiology Division, Dalla Lana School of Public Health, University of Toronto, Toronto, Canada; Epidemiology Division, Dalla Lana School of Public Health, University of Toronto, Toronto, Canada; Epidemiology Division, Dalla Lana School of Public Health, University of Toronto, Toronto, Canada; Child Health Evaluative Sciences, SickKids Research Institute, The Hospital for Sick Children, Toronto, Canada; Department of Health & Society, University of Toronto Scarborough, Scarborough, Canada; School of Medicine, University of California, Irvine, CA, USA; Stanford Prevention Research Center, School of Medicine , Stanford University, Stanford, CA, USA; Division of Preventive Medicine, Department of Family Medicine, UC San Diego, San Diego, CA, USA; Division of Preventive Medicine, Harvard Medical School, Boston, MA, USA; Department of Medicine, Brigham and Women’s Hospital, Boston, MA, USA; Department of Epidemiology, Harvard T.H. Chan School of Public Health, Boston, MA, USA; Department of Epidemiology, Fielding School of Public Health, School of Nursing, UCLA, Los Angeles, CA, USA; Obstetrics and Gynecology, University of Oklahoma, Oklahoma City, OK, USA; Epidemiology and Biostatistics Department, University of Arizona, Tucson, AZ, USA; Division of Geriatrics, Gerontology, and Palliative Care, Department of Medicine, Herbert Wertheim School of Public Health & Human Longevity Science, UC San Diego, San Diego, CA, USA; Department of Health Promotion Science, University of Arizona, Tucson, AZ, USA; Division of Preventive Medicine, Department of Family Medicine, UC San Diego, San Diego, CA, USA

**Keywords:** reproductive health, menarche, menopause, women’s health, postmenopausal, aging, obesity, adiposity, abdominal adiposity, body composition

## Abstract

**STUDY QUESTION:**

What is the association between reproductive health history (e.g. age at menarche, menopause, reproductive lifespan) with abdominal adiposity in postmenopausal women?

**SUMMARY ANSWER:**

Higher visceral adipose tissue (VAT) and subcutaneous adipose tissue (SAT) tissue levels were observed among women with earlier menarche, earlier menopause, and greater parity.

**WHAT IS KNOWN ALREADY:**

Postmenopausal women are predisposed to accumulation of VAT and SAT. Reproductive health variables are known predictors of overall obesity status in women, defined by BMI.

**STUDY DESIGN, SIZE, DURATION:**

This study is a secondary analysis of data collected from the baseline visit of the Women’s Health Initiative (WHI). The WHI is a large prospective study of postmenopausal women, including both a randomized trial and observational study. There were 10 184 women included in this analysis.

**PARTICIPANTS/MATERIALS, SETTING, METHODS:**

Data were collected from a reproductive health history questionnaire, dual-energy x-ray absorptiometry scans, and anthropometric measures at WHI baseline. Reproductive history was measured via self-report, and included age at menarche, variables related to pregnancy, and age at menopause. Reproductive lifespan was calculated as age at menopause minus age at menarche. Statistical analyses included descriptive analyses and multivariable linear regression models to examine the association between reproductive history with VAT, SAT, total body fat, and BMI.

**MAIN RESULTS AND THE ROLE OF CHANCE:**

Women who reported early menarche (<10 years) or early menopause (<40 years) had the highest levels of VAT. Adjusted multivariable linear regression results demonstrate women who experienced menarche >15 years had 23 cm^2^ less VAT (95% CI: −31.4, −14.4) and 47 cm^2^ less SAT (95% CI: −61.8, −33.4) than women who experienced menarche at age 10 years or earlier. A similar pattern was observed for age at menopause: compared to women who experienced menopause <40 years, menopause at 50–55 years was associated with 19.3 cm^2^ (95% CI: −25.4, −13.3) less VAT and 27.4 cm^2^ (−29.6, 10.3) less SAT. High parity (>3 pregnancies) was also associated with VAT and SAT. For example, adjusted beta coefficients for VAT were 8.36 (4.33, 12.4) and 17.9 (12.6, 23.2) comparing three to four pregnancies with the referent, one to two pregnancies.

**LIMITATIONS, REASONS FOR CAUTION:**

The WHI reproductive health history questionnaire may be subject to poor recall owing to a long look-back window. Residual confounding may be present given lack of data on early life characteristics, such as maternal and pre-menarche characteristics.

**WIDER IMPLICATIONS OF THE FINDINGS:**

This study contributes to our understanding of reproductive lifespan, including menarche and menopause, as an important predictor of late-life adiposity in women. Reproductive health has also been recognized as a sentinel marker for chronic disease in late life. Given established links between adiposity and cardiometabolic outcomes, this research has implications for future research, clinical practice, and public health policy that makes use of reproductive health history as an opportunity for chronic disease prevention.

**STUDY FUNDING/COMPETING INTEREST(S):**

HRB and AOO are supported by the National Institute of Health National Institute of Aging (R01AG055018-04). JWB reports royalties from ‘ACSM’S Body Composition Assessment Book’ and consulting fees from the WHI. The remaining authors have no competing interests to declare.

**TRIAL REGISTRATION NUMBER:**

N/A.

## Introduction

Substantial evidence demonstrates that accumulation of abdominal adipose tissue is strongly associated with chronic disease outcomes (Fox *et al.*, 2007; Porter *et al.*, 2009; Liu *et al.*, 2010; Britton *et al.*, 2013; Després and Tchernof, 2013; Shah *et al.*, 2014; Abraham *et al.*, 2015; Lalia *et al.*, 2016). Abdominal adipose tissue comprised visceral and subcutaneous adipose tissue (SAT). Visceral adipose tissue (VAT) is hormonally active fat that accumulates when SAT can no longer store nutrients (Després and Tchernof, 2013; Abraham *et al.*, 2015). VAT is hypothesized to be the driver of many health risks associated with excess adiposity via metabolic activity that appears to promote oxidative stress and an inflammatory state, and thus the downstream metabolic and cardiovascular health consequences (Tchkonia *et al.*, 2002; Britton and Fox, 2011; Després and Tchernof, 2013). Prior research demonstrates that SAT may be relatively less important as a predictor of cardiometabolic disease (Liu *et al.*, 2010). The distribution of adipose tissue is also recognized as a key component of cardiometabolic disease risk. Android fat accumulation (around the abdomen and midsection) is recognized as a greater risk factor than gynoid fat (around the hips and thighs) (Xu *et al.*, 2023). The type and distribution of adipose tissue are being increasingly recognized as key risk factors for cardiometabolic disease, even more so than traditional metrics such as BMI and waist circumference (Baarts *et al.*, 2023).

Given the extant literature highlighting the relationship between abdominal adipose tissue and cardiometabolic disease, there is a clear need for research focused on predictors of abdominal adiposity. Postmenopausal women are a uniquely high-risk group in this regard, as they have a propensity toward VAT accumulation (Pradhan, 2014). Prior studies have demonstrated an association between an increase in VAT and cardiometabolic disease risk during the menopausal transition, which has been attributed to decreased estrogens and increased androgens (Fox *et al.*, 2007; Porter *et al.*, 2009; Liu *et al.*, 2010). Estrogen acts in a cardioprotective fashion on vascular endothelial and smooth muscle cells to improve arterial response to injury, promote reendothelialization, inhibit matrix deposition, and prevent coronary artery vasospasm through vasodilation (Shah *et al.*, 2014; Lalia *et al.*, 2016). Decreased estrogen levels following menopause contribute to hypertension, increased carotid intima-media thickness, and coronary artery calcification (Tchkonia *et al.*, 2002; Lalia *et al.*, 2016). The change in hormone levels that occurs during the menopausal transition and postmenopausal period leads to dysregulation of lipid metabolism. This contributes to increased abdominal adiposity in postmenopausal women (Porter *et al.*, 2009). There has also been recent evidence of rising FSH as a correlate of increased adiposity in postmenopausal women (Mattick *et al.*, 2022).

Reproductive history is increasingly recognized as a risk factor for late-life cardiometabolic disease in women (Iorga *et al.*, 2017; Grandi *et al.*, 2019). However, the specific pathophysiologic mechanisms linking reproductive characteristics and cardiometabolic disease are unclear. Adiposity, including VAT accumulation, is a hypothesized risk factor in this causal pathway. Factors related to reproductive history, such as age at menarche, age at first birth, and parity, have been examined previously in relation to obesity measured by BMI or waist circumference (Trikudanathan *et al.*, 2013; Abraham *et al.*, 2015; Bubach *et al.*, 2016; Peters *et al.*, 2016; Pacyga *et al.*, 2019; Mishra *et al.*, 2021; Amiri *et al.*, 2023). In this manuscript, we will extend our current understanding of the relationship between reproductive characteristics and adiposity in postmenopausal women by leveraging technological advances that allow for estimation of VAT, SAT, android, and gynoid body fat distribution from dual-energy x-ray absorptiometry (DXA) scan (Bea *et al.*, 2022). Furthermore, we will also examine whether there are differences in the relationship between reproductive health history and abdominal adiposity by race/ethnicity. There are known differences in adiposity in women by race/ethnicity and evidence that the relationship between reproductive health and mortality differs in Black and White women (Grandi *et al.*, 2022). The objective of this study is to examine the association of reproductive history with novel measures of abdominal adipose tissue (VAT, SAT) and adipose tissue distribution in a racially and ethnically diverse sample of postmenopausal women.

## Materials and methods

### Study design and population

From 1993 to 1998, the Women’s Health Initiative (WHI) clinical trials and observational study recruited postmenopausal women aged 50–79 years. In total, 161 808 women were recruited from 40 clinical centers across the USA (Hays *et al.*, 2003). Three of the clinical centers were selected for a WHI sub-study on body composition; all WHI participants enrolled at the centers in Pittsburgh, PA (n = 3590); Birmingham, AL (n = 3665); and Tucson/Phoenix, AZ (n = 3765) were invited to participate (Chen *et al.*, 2007).

This manuscript describes a secondary analysis of data collected from 10 184 postmenopausal women who completed DXA scans at WHI baseline. At baseline, women were asked to recall variables related to early and mid-life reproductive health. All procedures and protocols were approved by the institutional review boards at each WHI participating institution and every participant provided written informed consent.

### Measures

#### Exposures

##### Reproductive health history

At study baseline, women were asked to complete a comprehensive questionnaire on their reproductive health history (Langer *et al.*, 2003; Murphy *et al.*, 2017). Women provided information on age at menarche (first menstrual period). Questionnaires collected detailed information on number of pregnancies and pregnancy-related history, including history of breastfeeding. Women were asked about miscarriage or difficulty conceiving (defined as ≥ 1 year attempting to become pregnant without conception), and for those who indicated difficulty conceiving, reasons for infertility (Kabat *et al.*, 2012). All WHI participants were postmenopausal, having had a hysterectomy or no menstrual bleeding for the previous 6 months [if age ≥56 years] or 12 months [if age 50–55 years]. Age at menopause was defined as the age at which participants experienced last menstrual bleeding, bilateral oophorectomy, or initiation of postmenopausal hormone therapy (Hays *et al.*, 2003; Langer *et al.*, 2003; Stefanick *et al.*, 2003). WHI also collected information on menopausal symptoms (e.g. hot flashes, night sweating) and use of postmenopausal hormone therapy (type, duration). Reproductive lifespan was calculated by subtracting age at menarche from age at menopause.

#### Outcomes

The primary outcome variables in this analysis are measures of adiposity from DXA scan (total body fat, percent body fat, visceral, and SAT) and anthropometric measures of adiposity. Outcome measures from WHI baseline visit were used in all analyses.

##### Measures from DXA

Whole-body scans were used to determine regional and total body composition using Hologic QDR2000 and 4500 W scanners. The DXA scans produced whole-body measurements of bone mineral density, lean mass, and fat mass (Chen *et al.*, 2005). Regional adiposity measurements were also available from DXA, enabling examination of fat mass in the trunk (neck, chest, abdominal area), pelvis, head, neck, arms, and legs. As part of the initial WHI study protocol, results from the DXA scans were used to assess total and regional amount of body fat in kilograms (kg) and percent fat (%), calculated as fat mass divided by body mass (Bea *et al.*, 2022). Manufacturer-defined regions of interest (ROI) were used to analyze DXA scans using QDR System software ver. 12.1 (Hologic I 2015). The WHI had comprehensive quality assurance plans including standardized protocols for positioning and analysis, technicians trained by Hologic, phantom scans for calibration by each site (spine, hip, and block calibration), and review of machine and technician performance.

Recently, archived WHI DXA scans were re-analyzed to obtain measures of abdominal VAT and SAT using Hologic APEX 4.0 software. As described in the Hologic Operator Manual, VAT and SAT were estimated in an abdominal ROI 5 cm in height across the full width of the abdomen at approximately the 4th lumbar vertebrae, while avoiding the iliac crest and limiting interference with soft tissue measures. To estimate VAT and SAT in the ROI, lines of demarcation in the abdominal area were aligned on both the outer edges of the soft tissue and the visceral cavity area. Algorithms were then used to generate values for VAT and SAT area (cm^2^): subtracting the derived SAT value from total abdominal adipose tissue estimate results in an estimate of VAT. The measurement approach for VAT and SAT has been validated in the WHI in comparison to MRI scans, demonstrating high validity and inter-/intra-rater reliability (Bea *et al.*, 2022).

Measures of body fat distribution (regional android and gynoid adiposity) were also obtained from Hologic Apex 4.0 software. The android region encompasses the lower portion of the torso, approximately defined as the area around the waist between the middle of the lumbar spine and top of the pelvis, immediately superior to iliac crests. The gynoid region encompasses the hip and upper thigh region, from the head of the femur to mid-thigh (Capers *et al.*, 2016). Individuals with high levels of android fat have an ‘apple’ shaped body fat distribution and those with a high level of gynoid fat are considered to be ‘pear’ shaped.

##### Anthropometric measures

At WHI baseline, trained examiners measured height, weight, and waist and hip circumferences using standardized protocols (Langer *et al.*, 2003). Weight was measured using a balance beam scale and height was measured using a wall-fixed stadiometer. BMI was calculated from measured height and weight as kilograms per meters squared (kg/m^2^). Waist circumference was measured using a tape measure at the midpoint between the last floating rib and the upper part of the iliac crest at end expiration.

#### Covariates

Data on all covariates were collected at WHI baseline. Self-report questionnaires were used to assess relevant covariates including age, annual family income, educational attainment, tobacco smoking (pack years), minutes of recreational physical activity per week. The WHI food frequency questionnaire was used to measure alcohol intake per week. Participants selected their race and ethnicity from researcher-defined categories. Owing to sample size restrictions, in the analysis, we examined three categories of race/ethnicity: non-Hispanic White, non-Hispanic Black, and Hispanic/Latina (of any race). We recognize that race in this context is a proxy for structural racism, defined as the structures and norms patterning societal inequality (Després and Tchernof, 2013; Abraham *et al.*, 2015).

### Statistical analysis

Descriptive statistics were used to examine mean VAT and SAT (area in cm^2^) and fat mass distribution (android, gynoid) by age at menarche, age at menopause, physical symptoms of menopause, bilateral oophorectomy, hysterectomy, number of pregnancies, history of miscarriage, and history of breastfeeding (>1 month). We further describe the relationship between variables related to self-report infertility or sub-fertility (defined as ≥ 1 year attempting to become pregnant without conception) with mean levels of VAT, SAT, and total body fat.

We then further investigated the role of age at menarche, age at menopause, and parity on late-life adiposity and body composition. Linear regression models were used to describe the crude and adjusted relationship between age at menarche (<10, 11, 12, 13, 14, >15 years), age at menopause (<40, 40–44, 45–50, 50–55, 55–60 years), and parity (0, 1–2, >3 pregnancies) with continuous measures of VAT, SAT, total body fat, android fat, gynoid fat, and BMI. Beta coefficients from a linear regression model are interpreted as the magnitude of change in the outcome per unit change in the exposure. As an example, for the VAT outcome, in models examining the exposure age at menarche (in years), the beta coefficient represents the change in VAT comparing women who experienced menarche at 11 years compared to <10 years, 12 years compared to <10 years, 13 years compared to <10 years, 14 years compared to <10 years, and 15 years compared to <10 years. In models examining age at menopause, again using VAT as an example, the beta coefficients represent the change in VAT among women who went through menopause between age 40–44 years compared to <40 years, age 45–50 years compared to <40 years, age 50–55 years compared to <40 years, and 55–60 years compared to compared to <40 years. Finally, for parity, the referent group was one to two pregnancies, so the beta coefficients are interpreted relative to this group (i.e. change in VAT for women who had one to two pregnancies compared to women with zero pregnancies, change in VAT for women who had three to four pregnancies compared to women with one to two pregnancies, and change in VAT for women who had five pregnancies compared to women with one to two pregnancies).

Analyses were adjusted for baseline covariates age, smoking status, race/ethnicity, income, education, WHI trial participation, healthy eating index (HEI) diet score, total energy intake, alcohol consumption, and physical activity level. We adjusted for WHI trial participation to account for differences in baseline characteristics between trial and observational study participants (Hays *et al.*, 2003; Langer *et al.*, 2003; Stefanick *et al.*, 2003). Confounder selection was completed based on theoretical knowledge of existing relationships and prior literature (Lee, 2014). In this analysis, we made the decision to adjust for diet score, energy intake, alcohol consumption, and physical activity level, despite the fact that they were measured after (i.e. temporally subsequent to) the exposure. VanderWeele’s recent guidance on principles for confounder selection in epidemiology advises for controlling for covariates that are a cause of the exposure, or outcome, or both to remove potential confounding effects (VanderWeele, 2019). This approach to confounder selection is called the disjunctive cause criterion (VanderWeele, 2019). As such, we adjusted for diet, alcohol, and physical activity level given their strong association with the outcome of interest (adiposity).

To assess whether there is a difference in adiposity level according to natural menopause or surgical menopause, we stratified models according to self-report bilateral oophorectomy status. There were 2010 women (20.5%) who reported having had a bilateral oophorectomy. Additionally, prior research in the WHI has demonstrated differences in adiposity according to race and ethnicity. We examined the relationship between age at menarche, age at menopause, and parity stratified by race/ethnicity (non-Hispanic White, non-Hispanic Black, Hispanic). In this stratified analysis, we categorized parity as one-two, three-four, or five live births owing to an insufficient sample size for women who had no term pregnancies.

Finally, we investigated the relationship between length of reproductive lifespan (years) and abdominal adiposity. To assess potential for interaction, we included product interaction terms for reproductive lifespan x race/ethnicity. Interaction terms were included in multivariable-adjusted linear regression models, including the same covariates as described above.

## Results

Among the 10 184 postmenopausal women in the sample, the majority (69%) had completed high school or equivalent, were married (62%), and had a household income between $35 000 and $75 000 (57%). On an average, women in the WHI DXA sub-cohort were 62.4 (SD ±7.4) years at baseline. Few women reported current smoking (8%), but 37% reported former smoking and 55% reported never smoking. Mean BMI was 28.2 (SD ±5.9) kg/m^2^ and waist circumference was 85.8 (SD ±13.4) cm. Consistent with WHO/CDC criteria, BMI was also examined as a categorical variable: 0.8% BMI <18.5, 32.0% BMI 18.5–24.9, 35.1% BMI 25–29.9, 19.8% BMI 30–34.9, 8.1% BMI 35–39.9, and 4.2% BMI >40 kg/m^2^. Additional information on demographic characteristics of the WHI DXA cohort is described in Supplementary Tables S1, S2, and S3, overall and stratified by age at menarche and parity. The majority of WHI participants reported beginning menses between 11 and 14 years of age (11 years: 14.9%, 12 years: 25.4%, 13 years: 28.5%, 14 years: 13.8%). 8.9% of women had never been pregnant, 2.3% never had a full-term pregnancy, 9.1% had one, 23.1% had two, 23.8% had three, 16.0% had four, and 16.8% had five or more full-term pregnancies. Average age at menopause was 47.5 years (SD = 6.9). Of the 10 184 women in the sample, 20.5% reported having a bilateral oophorectomy and 48.9% reported having a hysterectomy.

In descriptive analyses stratified by reproductive factors, women with a younger age at menarche had higher levels of VAT (cm^2^), SAT (cm^2^), android, and gynoid fat mass (kg) (Table 1). Similar relationships were observed for age at menopause. Women who experienced menopause early (<40 years) also had higher levels of VAT, SAT, android, and gynoid fat compared to women who underwent menopause after age 50 years (Table 1). Women who reported having bilateral oophorectomy or hysterectomy had higher levels of VAT and SAT than those who did not have either procedure. There was no consistent relationship between having any physical symptoms of menopause (e.g. hot flashes) and adiposity (Table 1). With regard to pregnancy-related variables, having a greater number of pregnancies resulted in greater VAT, SAT, and fat mass (one pregnancy had 163.2 cm^2^ VAT compared to 186.8 cm^2^ VAT for women with five or more pregnancies) (Table 1). There were no marked differences in adiposity variables across variables related to sub-fertility or infertility (Table 2). In women who reported reasons for infertility, VAT, android fat, and gynoid fat were higher among women who experienced infertility related to hormone dysregulation or ovulation (e.g. 13.7 cm VAT higher in women who reported infertility related to hormones) (Table 2).

**Table 1.** Comparison of women’s reproductive health history variables by visceral adipose tissue (VAT) area (cm^2^), subcutaneous adipose tissue (SAT) area (cm^2^), android, and gynoid fat (kg) (N = 10 184).

	VAT area (cm^2^)	SAT area (cm^2^)	Android fat mass (kg)	Gynoid fat mass (kg)
**Age at menarche (years)**				
≤9	191.9 (81.6)	456.4 (161.1)	3.11 (1.39)	6.44 (2.10)
10	178.5 (80.6)	409.6 (142.2)	2.76 (1.27)	5.87 (1.90)
11	175.9 (86.0)	399.7 (140.2)	2.71 (1.30)	5.96 (1.88)
12	167.5 (82.7)	383.4 (138.2)	2.55 (1.24)	5.64 (1.76)
13	159.9 (80.6)	369.1 (135.7)	2.43 (1.20)	5.48 (1.76)
14	161.1 (79.4)	366.6 (135.9)	2.42 (1.20)	5.47 (1.80)
15	164.7 (80.9)	367.7 (131.1)	2.42 (1.20)	5.36 (1.74)
16	163.4 (81.8)	374.8 (141.5)	2.47 (1.22)	5.47 (1.88)
>17	158.9 (82.8)	368.7 (141.5)	2.39 (1.20)	5.18 (1.60)
**Age at menopause**				
<40 years	182.6 (82.9)	408.3 (144.2)	2.78 (1.29)	5.89 (1.93)
40–50 years	163.7 (82.1)	378.0 (136.8)	2.50 (1.23)	5.64 (1.80)
>50 years	159.9 (80.2)	370.5 (136.2)	2.44 (1.21)	5.50 (1.76)
**Symptoms of menopause**				
No	167.7 (434.9)	377.5 (142.1)	2.52 (1.26)	5.55 (1.86)
Yes	163.7 (424.7)	379.9 (137.8)	2.51 (1.23)	5.61 (1.79)
**Bilateral oophorectomy**				
No	164.0 (82.2)	1963.1 (721.4)	2.50 (1.24)	5.57 (1.80)
Yes	170.3 (81.2)	2007.9 (705.4)	2.58 (1.22)	5.71 (1.78)
**Hysterectomy**				
No	159.7 (81.7)	369.2 (139.0)	2.43 (1.24)	5.49 (1.79)
Yes	172.8 (82.1)	391.4 (137.1)	2.63 (1.23)	5.72 (1.82)
**Number of full-term pregnancies**				
Never pregnant	157.8 (84.1)	368.3 (138.1)	2.42 (1.26)	5.51 (1.38)
No full-term pregnancies	163.2(82.8)	394.0 (142.4)	2.56 (1.30)	5.73 (1.79)
1	156.8 (80.5)	375.2 (146.2)	2.44 (1.27)	5.65 (1.90)
2	157.0 (81.1)	366.5 (135.6)	2.40 (1.21)	5.49 (1.78)
3	165.3 (81.5)	379.5 (137.3)	2.52 (1.24)	5.63 (1.75)
4	169.0 (79.2)	381.3 (133.7)	2.55 (1.19)	5.63 (1.80)
≥5	186.8 (83.1)	405.8 (140.6)	2.77 (1.26)	5.72 (1.82)
**History of miscarriage**				
No	164.7 (81.6)	378.9 (138.1)	2.51 (1.24)	5.61 (1.81)
Yes	168.1 (83.9)	382.2 (139.6)	2.55 (1.26)	5.64 (1.79)
**Breastfeeding (>1 month)**				
No	163.4 (81.9)	378.4 (138.7)	2.51 (1.26)	5.61 (1.83)
Yes	168.2 (82.3)	381.5 (138.7)	2.54 (1.22)	5.61 (1.79)

**Table 2.** Comparison of infertility and subfertility reproductive history and visceral adipose tissue (VAT) area (cm^2^), subcutaneous adipose tissue (SAT) area (cm^2^), and total body fat (kg) among postmenopausal women (N = 10 184).

	Visceral adipose tissue area (cm^2^)	Subcutaneous adipose tissue area (cm^2^)	Android fat mass (kg)	Gynoid fat mass (kg)
**>1 year to conceive**				
No	166.4 (82.6)	380.9 (139.2)	2.53 (1.25)	5.61 (1.82)
Yes	163.1 (79.7)	374.3 (135.0)	2.48 (1.19)	5.58 (1.72)
**Visit doctor or clinic because you did not become pregnant**				
No	167.9 (80.7)	384.5 (138.2)	2.56 (1.21)	5.66 (1.82)
Yes	161.3 (80.1)	370.9 (134.6)	2.45 (1.19)	5.53 (1.69)
**Was a reason found for infertility**				
No	162.6 (83.1)	370.4 (137.9)	2.47 (1.23)	5.58 (1.74)
Yes	160.3 (80.1)	369.5 (132.5)	2.43 (1.18)	5.50 (1.67)
**Reasons for infertility:**				
**Related to hormones or ovulation**				
No	158.0 (78.0)	396.0 (168.9)	2.37 (1.12)	5.44 (1.63)
Yes	171.7 (95.0)	380.7 (138.8)	2.71 (1.51)	5.83 (1.90)
**Related to uterus or fallopian tubes**				
No	157.5 (81.0)	365.4 (132.2)	2.38 (1.18)	5.41 (1.66)
Yes	164.0 (80.6)	375.7 (136.2)	2.49 (1.21)	5.64 (1.70)
**Related to endometriosis**				
No	162.2 (82.2)	371.7 (137.6)	2.45 (1.22)	5.52 (1.70)
Yes	149.0 (72.8)	357.8 (111.7)	2.27 (0.99)	5.42 (1.57)
**Related to partner**				
No	159.9 (78.7)	368.2 (133.9)	2.42 (1.19)	5.49 (1.70)
Yes	160.5 (85.7)	372.3 (133.7)	2.43 (1.20)	5.52 (1.65)

In multivariable linear regression analyses, women with a younger age at menarche had higher levels of adiposity, including VAT, SAT, total body fat, android fat, gynoid fat, and BMI (Table 3). The relationship appeared consistent across outcomes (i.e. different measures of adiposity) and monotonic as each successive year of menarche is associated with less adiposity up to age 15 years. For instance, compared to women who experienced menarche at 10 years or younger, women who had their first menstrual period at 11, 12, 13, 14, or 15 years had less visceral fat (4.7, 14.3, 21.7, 23.0, and 23.0 cm^2^, respectively) and subcutaneous fat (18.6, 32.6, 44.4, 49.4, 47.6 cm^2^, respectively) (Table 3). There was evidence of a dose–response decrease in both android and gynoid fat distribution in women according to age at menarche (Table 3). Early menopause (<40 years of age) was associated with higher adiposity across all measures of adiposity in adjusted linear regression models (Table 4). This relationship appeared monotonically decreasing except for a small increase in the highest category of age at menopause (55–60 years) (Table 4). In multivariable linear regression models examining parity and body composition, women with no pregnancies or >3 pregnancies had higher VAT, SAT, total body fat, android fat, gynoid fat, and BMI (Table 5). Finally, we examined regression models for the relationship between VAT and SAT with age at menarche, age at menopause, and parity stratified by self-report category race/ethnicity (Table 6).

**Table 3.** Linear regression results examining the relationship of age at menarche with measures of body composition among postmenopausal women (N = 10 184).

Outcome	Age (years)	**Crude** β **(95% CI)**	**Adjusted** * β **(95% CI)**
VAT (cm^2^)	<10	−ref-	−ref-
	11	0 − 5.6 (−13.3, −2.10)	0 − 4.7 (−12.8, 3.33)
	12	−14.0 (−21.3, −6.75)	−14.3 (−21.9, −6.68)
	13	−21.6 (−28.8, −14.4)	−21.7 (−29.2, −14.1)
	14	−20.4 (−28.2, −12.6)	−23.0 (−31.3, −14.8)
	>15	−17.8 (−25.9, −9.81)	−23.0 (−31.4, −14.4)
SAT (cm^2^)	<10	−ref-	−ref-
	11	−20.4 (−72.1, 11.0)	−18.6 (−32.0, −5.15)
	12	−36.7 (−117.9, −39.6)	−32.6 (−45.3, −19.9)
	13	−51.0 (−152.1, −74.6)	−44.4 (−57.3, −32.1)
	14	−53.5 (−163.7, 79.3)	−49.4 (−63.0, −35.7)
	>15	−49.9 (−161.3, 73.9)	−47.6 (−61.8, −33.4)
Total body fat (kg)	<10	−ref-	−ref-
	11	−0.18 (−0.45, 0.1)	−0.18 (−0.47, 0.10)
	12	−0.65 (−0.91, −0.40)	−0.60 (−0.87, −0.33)
	13	−0.93 (−1.19, −0.68)	−0.86 (−1.13, −0.60)
	14	−0.95 (−1.23, −0.68)	−0.87 (−1.16, −0.58)
	>15	−1.03 (1.32, −0.75)	−1.00 (−1.30, −0.70)
Android fat mass (kg)	<10	−ref-	−ref-
	11	−0.13 (−0.25, −0.014)	−0.12 (−0.24, 0.27)
	12	−0.29 (−0.40, −0.18)	−0.26 (−0.38, −0.15)
	13	−0.41 (−0.52, −0.30)	−0.37 (−0.49, −0.26)
	14	−0.42 (−0.54, −0.30)	−0.41 (−0.53, −0.28)
	>15	−0.41 (−0.53, −0.29)	−0.41 (−0.54, −0.29)
Gynoid fat mass (kg)	<10	−ref-	−ref-
	11	−0.45 (−0.21, 0.12)	−0.59 (−0.24, 0.12)
	12	−0.37 (−0.53, −0.21)	−0.34 (−0.51, −0.171)
	13	−0.52 (−0.68, −0.36)	−0.49 (−0.65, −0.32)
	14	−0.53 (−0.70, −0.36)	−0.46 (−0.64, −0.28)
	>15	−0.62 (−0.80, −0.45)	−0.59 (−0.77, −0.40)
BMI (kg/m^2^)	<10	−ref-	−ref-
	11	−1.14 (−1.69, −0.59)	−1.17 (−1.74, −0.56)
	12	−1.96 (−2.48, −1.45)	−1.88 (−2.43, −1.34)
	13	−2.67 (−3.18, −2.15)	−2.56 (−3.09, −2.02)
	14	−2.81 (−3.37, −2.25)	−2.85 (−3.43, −2.27)
	>15	−2.70 (−3.28, −2.13)	−2.86 (−3.46, −2.26)

* Models adjusted for age, smoking status, race/ethnicity, WHI trial participation, diet score, total energy intake, alcohol intake, physical activity, income, education.

VAT, visceral adipose tissue; SAT, subcutaneous adipose tissue; Ref, reference category; β, beta coefficient.

**Table 4.** Linear regression results examining the relationship of age at menopause with measures of body composition among postmenopausal women (N = 10 184).

				Stratified results	
Outcome	Age (years)	Crude β (95% CI)	**Adjusted** * **β (95% CI)**	No bilateral oophorectomy	Bilateral oophorectomy
VAT (cm^2^)	<40	−ref-	−ref-	−ref-	−ref-
	40–44	−12.8 (−19.3, −6.4)	−12.1 (−18.9, −5.4)	−11.1 (−20.6, −2.65)	−10.4 (−21.0, 0.18)
	45–50	−22.6 (−28.5, −16.8)	−18.8 (−25.0, −12.5)	−20.7 (−29.4, −12.0)	−4.80 (−15.2, 5.6)
	50–55	−25.0 (−30.6, −19.4)	−19.3 (−25.4, −13.3)	−17.6 (−25.9, −9.2)	−16.2 (−27.6, −4.8)
	55–60	−16.9 (−23.5, −10.2)	−13.3 (−20.5, −6.09)	−11.5 (−20.8, −2.1)	−7.2 (−27.2, 12.8)
SAT (cm^2^)	<40	−ref-	−ref-	−ref-	−ref-
	40–44	−23.2 (−34.0, −12.3)	−19.2 (−27.5, −5.13)	−12.9 (−28.8, 3.05)	−23.3 (−40.7, −5.9)
	45–50	−34.6 (−44.5, −24.7)	−27.1 (−33.1, −12.6)	−26.1 (−40.7, −11.4)	−12.4 (−29.5, 4.7)
	50–55	−40.0 (−49.5, −30.5)	−27.4 (−29.6, −10.3)	−21.5 (−35.7, −7.4)	−26.8 (−45.6, −8.2)
	55–60	−32.0 (−43.3, −20.8)	−10.6 (−16.1, 7.48)	0 − 4.7 (−20.4, 11.0)	−15.2 (−48.0, 17.6)
Total body fat (kg)	<40	−ref-	−ref-	−ref-	−ref-
	40–44	−0.41 (−0.64, −0.19)	−0.32 (−0.56, −0.08)	−0.22 (−0.56, 0.11)	−0.36(−0.73, 0.01)
	45–50	−0.61 (−0.81, −0.40)	−0.48 (−0.70, −0.26)	−0.51 (−0.82, 0.20)	−0.05 (−0.42, 0.31)
	50–55	−0.76 (−0.96, −0.57)	−0.52 (−0.73, −0.31)	−0.43 (−0.73, −0.13)	−0.44 (−0.84, −0.04)
	55–60	−0.64 (−0.88, −0.41)	−0.17 (−0.43, 0.08)	0.08 (−0.41, 0.25)	−0.14 (−0.84, −0.60)
Android fat (kg)	<40	−ref-	−ref-	−ref-	−ref-
	40–44	−0.21 (−0.31, −0.12)	−0.18 (−0.28, −0.08)	−0.14 (−0.28, 0.007)	−0.20 (−0.35, −0.32)
	45–50	−0.31 (−0.40, −0.23)	−0.25 (−0.35, −0.16)	−0.26 (−0.39, −0.13)	−0.79 (−0.24, 0.08)
	50–55	−0.36 (−0.45, −0.28)	−0.26 (−0.35, −0.17)	−0.21 (−0.34, −0.09)	−0.24 (−0.41, −0.69)
	55–60	−0.29 (−0.39, −0.19)	−0.12 (−0.23, −0.02)	−0.08 (−0.22, 0.06)	−0.10 (−0.40, 0.20)
Gynoid fat (kg)	<40	−ref-	−ref-	−ref-	−ref-
	40–44	−0.20 (−0.34, −0.06)	−0.14 (−290.7, 10.4)	−0.09 (−0.30, 0.12)	−0.17 (−0.40, 0.07)
	45–50	−0.29 (−0.41, −0.16)	−0.22 (−362.7, −85.4)	−0.25 (−0.44, −0.05)	0.024 (−207.6, 0.26)
	50–55	−0.40 (−0.52, −0.28)	−0.26 (−393.9, −125.7)	−0.22 (−0.41, −0.04)	−0.20 (−0.45, 0.05)
	55–60	−0.35 (−0.50, −0.21)	−0.05 (−0.21, 0.11)	−0.002 (−0.21, 0.21)	−0.04 (−0.48, 0.41)
BMI (kg/m^2^)	<40	−ref-	−ref-	−ref-	−ref-
	40–44	−0.79 (−1.25, −0.31)	−0.60 (−1.09, −0.11)	−0.53 (−1.21, 0.15)	−0.45 (1.21, 0.32)
	45–50	−1.31 (−1.73, −0.89)	−0.94 (−1.39, −0.49)	−1.04 (−1.67, −0.42)	0.06 (−0.69, 0.81)
	50–55	−1.49 (−1.89, −1.09)	−0.93 (−1.37, −0.50)	−0.80 (−1.40, −0.19)	−0.54 (−1.36, 0.28)
	55–60	−1.12 (−1.60, −0.64)	−0.30 (−0.82, 0.21)	−0.18 (−0.85, 0.49)	0.29 (−1.15, 1.73)

* Models adjusted for age, smoking status, race/ethnicity, WHI trial participation, hormone therapy use, diet score, total energy intake, alcohol intake, physical activity, income, education, parity, sub-fertility.

VAT, visceral adipose tissue; SAT, subcutaneous adipose tissue; Ref, reference category; β, beta coefficient.

**Table 5.** Linear regression results examining the relationship of parity (number of full-term pregnancies) with measures of adiposity among postmenopausal women (N = 10 184).

Outcome	**Parity** *	**Crude** β **(95% CI)**	**Adjusted** ** β **(95% CI)**
VAT (cm^2^)	0	6.26 (−4.47, 17.0)	2.28 (−9.19, 13.7)
	1–2	−ref-	−ref-
	3–4	9.65 (5.91, 13.4)	8.36 (4.33, 12.4)
	5	29.8 (25.1, 34.6)	17.9 (12.6, 23.2)
SAT (cm^2^)	0	25.1 (6.91, 43.4)	21.4 (2.19, 40.6)
	1–2	−ref-	−ref-
	3–4	11.1 (4.74, 17.4)	11.8 (5.05, 18.5)
	5	37.0 (28.9, 45.1)	19.1 (10.2, 27.9)
Total body fat (kg)	0	0.34 (−0.04, 0.73)	0.31 (−0.10, 0.71)
	1–2	−ref-	−ref-
	3–4	0.21 (0.08, 0.34)	0.22 (0.08, 0.37)
	5	0.55 (0.38, 0.72)	0.33 (0.15, 0.52)
Android fat (Kg)	0	0.15 (−0.013, 0.31)	0.10 (−0.07, 0.27)
	1–2	−ref-	−ref-
	3–4	0.12 (0.07, 0.18)	0.12 (−0.06, 0.18)
	5	0.36 (0.28, 0.43)	0.21 (0.13, 0.29)
Gynoid fat (Kg)	0	0.19 (−0.05, 0.43)	0.20 (−0.04, 0.46)
	1–2	−ref-	−ref-
	3–4	0.09 (0.04, 0.17)	0.10 (0.02, 0.19)
	5	0.19 (0.08, 0.29)	0.12 (0.04, 0.24)
BMI (kg/m^2^)	0	0.98 (0.20, 1.76)	0.57 (−0.25, 1.40)
	1–2	−ref-	−ref-
	3–4	0.35 (0.08, 0.62)	0.27 (−0.02, 0.56)
	5	1.82 (1.5, 2.16)	0.96 (0.58, 1.34)

* There were 236 women who had no term pregnancies, 3263 who had 1–2 pregnancies, 4090 who had 3–4 pregnancies, and 1698 women who had five pregnancies.

** Models adjusted for age, smoking status, race/ethnicity, WHI trial participation, hormone therapy use, diet score, total energy intake, alcohol intake, physical activity, income, education, parity, sub-fertility.

VAT, visceral adipose tissue; SAT, subcutaneous adipose tissue; Ref, reference category; β, beta coefficient.

**Table 6.** Adjusted linear regression results comparing visceral adipose tissue (VAT) and subcutaneous adipose tissue (SAT) levels by age at menarche, age at menopause, and parity for non-Hispanic white, non-Hispanic black, and Hispanic women.

		Non-Hispanic White	Non-Hispanic Black	Hispanic
		β (95% CI)*	β (95% CI)*	β (95% CI)*
**N**		7828	1423	703
**VAT (cm^2^)**				
Mean, SD		161.6, 83.5	177.4, 72.5	185.3, 77.0
Age at menarche	<10	−ref-	−ref-	−ref-
	11	−3.20 (−12.7, 6.3)	−11.8 (−30.9, 7.42)	−2.14 (−33.9, 29.6)
	12	−12.8 (−21.8, −3.8)	−17.2 (−35.2, 0.87)	−32.6 (−62.6, −2.64)
	13	−21.7 (−30.6, −12.9)	−19.4 (−37.5, −1.28)	−28.8 (−58.8, 1.23)
	14	−23.6 (−33.2, −13.9)	−20.4 (−39.7, −1.10)	−27.3 (−59.3, 4.79)
	>15	−20.8 (−30.9, −10.7)	−27.7 (−47.1, −8.23)	−34.1 (−67.8, −0.38)
Age at menopause	<40	−ref-	−ref-	−ref-
	40–44	−13.0 (−21.0, −4.98)	−6.25 (−20.5, 8.03)	−6.39 (−33.6, 20.8)
	45–50	−21.0 (−28.3, −13.7)	−11.6 (−25.1, 1.94)	−3.96 (−29.1, 21.2)
	50–55	−19.2 (−26.3, −12.1)	−17.8 (−30.8, −4.71)	−21.8 (−46.0, 2.47)
	55–60	−15.1 (−23.5, −6.68)	−6.81 (−23.3, 9.66)	2.54 (−25.9, 30.9)
Parity	1–2	−ref-	−ref-	−ref-
	3–4	7.47 (2.98, 12.0)	10.6 (−0.03, 21.3)	20.9 (2.50, 39.4)
	5	15.7 (9.43, 21.9)	19.7 (7.48, 31.9)	43.5 (23.5, 63.5)
**SAT (cm^2^)**				
Mean, SD		363.5, 131.6	452.7, 151.5	402.7, 127.5
Age at menarche	<10	−ref-	−ref-	−ref-
	11	−19.1 (−34.1, −4.11)	−16.4 (−55.8, 23.0)	−12.4 (−66.0, 41.3)
	12	−34.8 (−48.8, −20.6)	−17.6 (−54.6, 19.4)	−43.4 (−94.1, 7.32)
	13	−48.3 (−61.4, −34.2)	−27.3 (−64.5, 9.85)	−34.9 (−85.7, 15.8)
	14	−53.9 (−69.5, −38.6)	−34.5 (−74.0, 5.07)	−42.0 (−96.2, 12.2)
	>15	−49.8 (−65.7, −33.8)	−43.2 (−83.1, −3.33)	−48.1 (−105.1, 8.9)
Age at menopause	<40	−ref-	−ref-	−ref-
	40–44	−17.7 (−30.5, −4.91)	−9.09 (−38.0, 19.8)	−25.1 (−70.6, 20.4)
	45–50	−24.7 (−36.4, −13.0)	−26.8 (−54.1, 0.60)	1.38 (−40.6, 43.4)
	50–55	−20.7 (−32.0, −9.39)	−29.5 (−56.0, −3.09)	−38.7 (−79.2, 1.83)
	55–60	−8.03 (−21.4, 5.38)	5.56 (−27.7, 38.9)	−16.8 (−64.2, 30.7)
Parity	1–2	−ref-	−ref-	−ref-
	3–4	13.8 (6.61, 20.9)	12.9 (−8.893, 34.7)	24.7 (−6.25, 55.7)
	5	15.9 (5.99, 25.9)	18.2 (−6.81, 43.2)	61.2 (35.6, 94.8)

* Models adjusted for age, smoking status, race/ethnicity, WHI trial participation, diet score, total energy intake, alcohol intake, physical activity, income, education.

Ref, reference category; β, beta coefficient.

**Figure 1.**
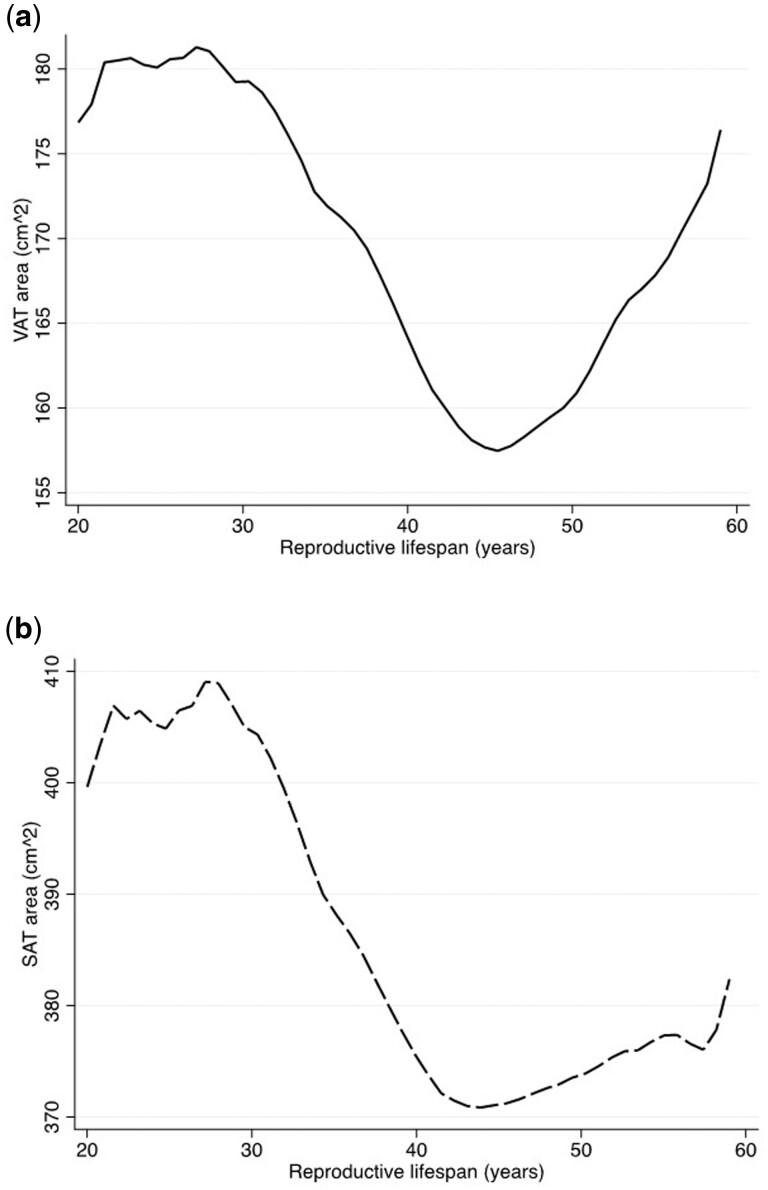
Visceral adipose tissue (VAT) and subcutaneous adipose tissue (SAT) area (cm^2^) by according to reproductive lifespan (years).

**Figure 2.**
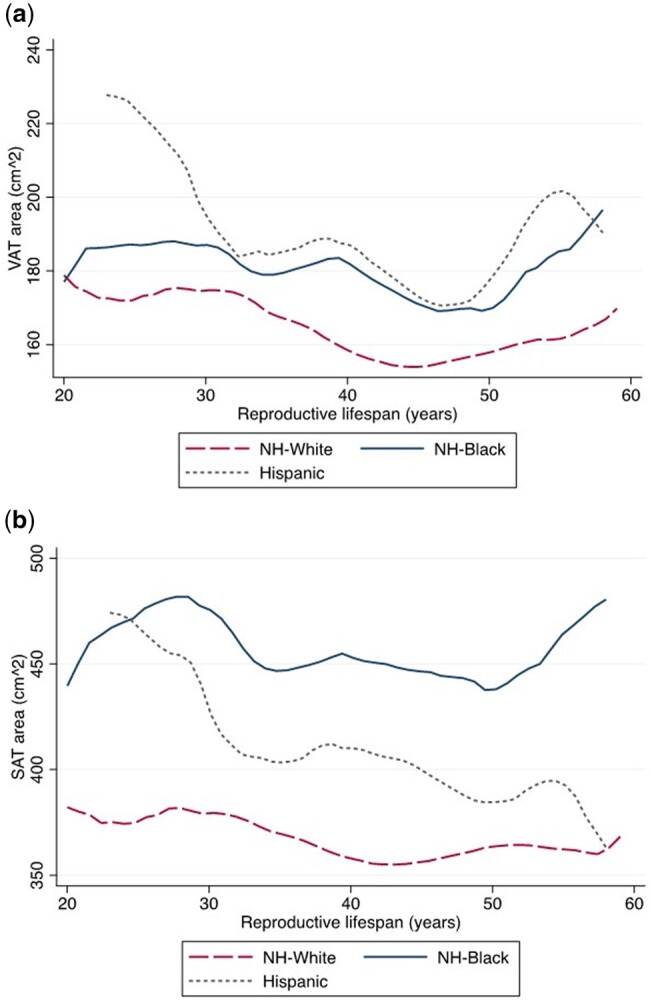
Visceral adipose tissue (VAT) and subcutaneous adipose tissue (SAT) area by reproductive lifespan (years) for non-Hispanic white, non-Hispanic black, and Hispanic women. NH, non-Hispanic.

Reproductive lifespan is the length of time from menarche to menopause. The average reproductive lifespan for all participants was 42.8 years (SD = 6.9, range: 14 to 59 years). As depicted in Fig. 1a and b, women with short reproductive lifespan had the highest levels of VAT; among women who had a reproductive span between 30- and 45-year VAT decreased and then increased again among those with a reproductive lifespan greater than 45 years (Fig. 1a). The relationship between SAT and reproductive lifespan shows a similar pattern, but without the same sharp increase at greater values for reproductive lifespan (Fig. 1b). Results examining the relationship between reproductive lifespan with VAT and SAT according to race/ethnicity are illustrated in Fig. 2a and b. There are differences in the quantity of VAT and SAT and total body fat and BMI according to length of reproductive lifespan for non-Hispanic white, non-Hispanic Black, and Hispanic women (Fig. 2; Supplementary Fig. S1). Non-Hispanic white women had the lowest levels of VAT and SAT regardless of reproductive lifespan, while Hispanic women appeared to have the greatest change in VAT and SAT associated with reproductive lifespan. As described in Table 6, the magnitude of the change in VAT across categories of age at menarche, age at menopause, and parity was largest among Hispanic women, followed by non-Hispanic black women and then non-Hispanic white women.

## Discussion

Results from the WHI demonstrate that postmenopausal women who experience early age at menarche or menopause, high parity, and shorter reproductive lifespan had increased levels of one or more of abdominal adiposity, most notably higher levels of VAT and android fat deposits. This study makes an important contribution to our understanding by describing the relationship between reproductive history with abdominal adiposity. The present results specifically highlight the impact of reproductive factors on the type and distribution of abdominal adipose tissue. This is an important contribution given the known health risks associated with VAT and excess android fat mass.

Existing literature has demonstrated associations between early menarche, early menopause, and increased parity with obesity defined by BMI (Trikudanathan *et al.*, 2013; Abraham *et al.*, 2015; Bubach *et al.*, 2016; Peters *et al.*, 2016; Mishra *et al.*, 2021; Amiri *et al.*, 2023). There is substantial evidence of a relationship between obesity and cardiovascular disease risk, diabetes, cancer, and mortality in women (Zajacova and Ailshire, 2014; Arnold *et al.*, 2016; Dhana *et al.*, 2016; Banack *et al.*, 2022). Taken together, this information demonstrates that adiposity may be a putative mediator between reproductive health history and morbidity and mortality in postmenopausal women. The novel DXA-derived indices of abdominal adiposity presented in this manuscript add to our understanding of these complex relationships. The quantity and patterning of adipose tissue have important health implications: visceral fat and high android fat mass are associated with greater cardiometabolic complications than subcutaneous fat or gynoid fat mass (Ma *et al.*, 2023). These results could have broad implications for primary prevention of chronic disease risk in postmenopausal women because visceral fat is a modifiable risk factor via diet, exercise, and medication (Gepner *et al.*, 2018; Wilding *et al.*, 2021).

In this study, women who experienced early menarche and/or early menopause had the highest levels of VAT. Given our knowledge of the relationship between VAT and cardiometabolic disease (Tchkonia *et al.*, 2002; Britton and Fox, 2011; Després and Tchernof, 2013), women with early menarche or menopause may represent a uniquely high-risk group. In particular, additional disease screening and follow-up may be indicated for women who experience early menarche and early menopause. The combination of early menarche and early menopause may indicate a phenotype at increased risk of cardiometabolic disease due, in part, to increased visceral adiposity. Android fat mass deposition is another particularly high-risk phenotype; central adiposity is associated with metabolic abnormalities, chronic disease, and mortality risk (Ma *et al.*, 2023). Women who experienced early menarche and/or early menopause also had highest levels of android fat. Reproductive health history has historically been underutilized in clinical and public health settings as a predictor of chronic disease development (Rich-Edwards, 2009; Mishra, 2010; Lancet, 2023). Further research is warranted that explores how information on age at menarche and age at menopause may be incorporated into preventive medicine and aging frameworks evaluating cardiometabolic risk in older women.

Moreover, our results also demonstrate a relationship between number of pregnancies (parity) and abdominal adiposity. Women who had one to two pregnancies had lower levels of VAT compared to women who had three, four, or five pregnancies. Prior work has demonstrated the importance of obstetrical history for cardiovascular disease prevention (Brown *et al.*, 2018; Agarwala *et al.*, 2020; Parikh *et al.*, 2021). In much the same way that women with a short reproductive lifespan may represent a high-risk phenotype for cardiometabolic disease via accumulation of VAT, women with high parity should also be considered potentially at-risk. These findings also parallel the American Heart Association’s statement on the potential for cardiovascular disease prevention among women who experience adverse pregnancy outcomes (Parikh *et al.*, 2021).

The relationship between parity and abdominal adiposity warrants further investigation. Parity is associated with higher BMI; both pre- and post-pregnancy BMI are higher in multiparous women, including long-term weight gain. Research from a large Danish cohort demonstrated mean BMI increased from 23.8 kg/m^2^ for women with one child to 26.7 kg/m^2^ for women with >5 pregnancies; corresponding with an increase of 0.62 kg/m^2^ (BMI units) per additional pregnancy (Iversen *et al.*, 2018). Our results indicate a stepwise increase in measures of adiposity, including VAT, SAT, and android fat mass according to parity, which is broadly consistent with prior research. Interestingly, there was minimal effect of parity on gynoid fat mass.

The present study did not find an increased level of adipose tissue among postmenopausal women who reported infertility or sub-fertility, in contrast to prior literature that suggests that women with obesity and other comorbidities are more likely to experience infertility or subfertility (Lau *et al.*, 2022; Murugappan *et al.*, 2022; Farland *et al.*, 2023). Cohort effects related to the WHI sample population or long recall periods (∼30–50 years) may have led to a lower reporting of infertility history. However, in the sub-group of women who reported infertility specifically related to hormone dysregulation or ovulation, there was an increase in adipose tissue levels. This is consistent with the literature on the relationship between hormone regulation and obesity in reproductive endocrine conditions such as polycystic ovary syndrome (Sam and Dhillo, 2010; He *et al.*, 2018; Amiri *et al.*, 2020; Cena *et al.*, 2020).

In analyses stratified by race/ethnicity, Hispanic women and non-Hispanic black women had higher levels of VAT and SAT than non-Hispanic white women. These findings are consistent with prior research in the WHI (Banack *et al.*, 2023) and with the broader literature on racial and ethnic differences in levels of adiposity in American women as a function of reproductive factors (Davis *et al.*, 2009). Prior research has demonstrated that young girls who are African-American experience an earlier age at menarche than those who are White. Early age at menarche has been associated with adult BMI in several studies, including an analysis linking age at menarche and high adult BMI using Mendelian randomization and a longitudinal analysis of nearly 50 years of prospective follow-up from a birth cohort study (Pierce and Leon, 2005; Gill *et al.*, 2018). Potential biological and psychosocial theories underlying this disparity include the intrauterine environment, early-life resources availability, and childhood BMI (Reagan *et al.*, 2012). Parous women who are African American or Hispanic have higher rates of parity-related weight gain than White women and racial disparities in obesity and weight gain in the perinatal period have been reported to persist throughout the life course for women (Davis *et al.*, 2009). Increased levels of visceral adiposity in Black and Hispanic women in our study represent an important finding for screening and risk stratification, highlighting the need for effective interventions in these groups.

The present study has several strengths. Firstly, the study utilized a validated approach for obtaining measures of abdominal adiposity from DXA scans in a large and diverse sample of postmenopausal women. Comprehensive data on reproductive history were collected as part of the WHI, an established longitudinal cohort of postmenopausal women. Owing to the size of the WHI cohort, we were able to assess potential interaction by race/ethnicity and examine stratified results among non-Hispanic black, non-Hispanic white, and Hispanic women. There are also some limitations to note. The WHI was initially designed as a prospective study of chronic disease outcomes in postmenopausal women. The study only collected basic information on pregnancy-related variables such as breastfeeding. Given this manuscript describes a secondary analysis of the WHI data, we are limited by the data that was previously collected. The WHI reproductive health history questionnaire may be subject to measurement error due to recall bias given the length of time between reproductive health exposures and time of data collection. However, self-reported reproductive history has been validated in prior research and has been shown to be fairly accurate (Harville *et al.*, 2019; Jung *et al.*, 2021). Misclassification or measurement error may be present given crude and self-report measures of reproductive health history (e.g. breastfeeding > or <1 month). Additionally, residual confounding may be present given the lack of measures of body weight or adiposity prior to menarche or throughout the reproductive time period, nor measures of early life socioeconomic or psychosocial variables that may impact late-life reproductive health and adiposity (Mishra *et al.*, 2009).

This study contributes to our understanding of reproductive health history and abdominal adiposity in postmenopausal women. Future research should examine whether specific reproductive health events (e.g. early menarche) represent sentinel health events or present an opportunity for risk stratification. Greater understanding of the downstream (late-life) clinical consequences of reproductive health characteristics would be a tremendous step forward for women’s health. The present study has implications for research, clinical practice, and public health policy in the areas of women’s health and aging. Our results represent an important opportunity to promote healthy aging and prevent chronic disease among postmenopausal women through harnessing reproductive health history.

## Supplementary Material

deae118_Supplementary_Figure_S1

deae118_Supplementary_Table_S1

deae118_Supplementary_Table_S2

deae118_Supplementary_Table_S3

## Data Availability

The data underlying this article will be shared on reasonable request to the corresponding author.

## References

[deae118-B1] Abraham TM , Pedley A, Massaro JM, Hoffmann U, Fox CS. Association between visceral and subcutaneous adipose depots and incident cardiovascular disease risk factors. Circulation 2015;132:1639–1647. 26294660 10.1161/CIRCULATIONAHA.114.015000 PMC4779497

[deae118-B3] Agarwala A , Michos ED, Samad Z, Ballantyne CM, Virani SS. The use of sex-specific factors in the assessment of women’s cardiovascular risk. Circulation 2020;141:592–599. 32065772 10.1161/CIRCULATIONAHA.119.043429 PMC7032610

[deae118-B4] Amiri M , Mousavi M, Azizi F, Ramezani Tehrani F. The relationship of reproductive factors with adiposity and body shape indices changes overtime: findings from a community-based study. J Transl Med 2023;21:137. 36814308 10.1186/s12967-023-04000-1 PMC9948339

[deae118-B5] Amiri M , Ramezani Tehrani F, Rahmati M, Firouzi F, Azizi F. Do trends of adiposity and metabolic parameters vary in women with different ovarian reserve status? A population-based cohort study. Menopause 2020;27:684–692. 32301892 10.1097/GME.0000000000001513

[deae118-B6] Arnold M , Jiang L, Stefanick ML, Johnson KC, Lane DS, LeBlanc ES, Prentice R, Rohan TE, Snively BM, Vitolins M et al Duration of adulthood overweight, obesity, and cancer risk in the Women’s Health Initiative: a longitudinal study from the United States. PLoS Med 2016;13:e1002081. 27529652 10.1371/journal.pmed.1002081 PMC4987008

[deae118-B7] Baarts RB , Jensen MR, Hansen OM, Haddock B, Prescott E, Hovind P, Simonsen L, Bülow J, Suetta C. Age- and sex-specific changes in visceral fat mass throughout the life-span. Obesity (Silver Spring) 2023;31:1953–1961. 37312268 10.1002/oby.23779

[deae118-B8] Banack HR , Bea JW, Chen Z, Blew RM, Nicholas S, Stefanick M, Wild RA, Manson JE, Odegaard AO. Longitudinal patterns of abdominal visceral and subcutaneous adipose tissue, total body composition, and anthropometric measures in postmenopausal women: results from the Women’s Health Initiative. Int J Obes (Lond) 2023;47:288–296. 36739471 10.1038/s41366-023-01266-9

[deae118-B9] Banack HR , Chang J, Stefanick ML, Arnold M, Anton-Culver H, Jiang L. Relationship between BMI trajectories and cardiometabolic outcomes in postmenopausal women: a growth mixture modeling approach. Ann Epidemiol 2022;72:9–17. 35469929 10.1016/j.annepidem.2022.04.004

[deae118-B10] Bea JW , Chen Z, Blew RM, Nicholas JS, Follis S, Bland VL, Cheng TD, Ochs-Balcom HM, Wactawski-Wende J, Banack HR et al MRI based validation of abdominal adipose tissue measurements from DXA in postmenopausal women. J Clin Densitom 2022;25:189–197. 34404568 10.1016/j.jocd.2021.07.010 PMC8799761

[deae118-B11] Britton KA , Fox CS. Ectopic fat depots and cardiovascular disease. Circulation 2011;124:e837–e841. 22156000 10.1161/CIRCULATIONAHA.111.077602

[deae118-B12] Britton KA , Massaro JM, Murabito JM, Kreger BE, Hoffmann U, Fox CS. Body fat distribution, incident cardiovascular disease, cancer, and all-cause mortality. J Am Coll Cardiol 2013;62:921–925. 23850922 10.1016/j.jacc.2013.06.027 PMC4142485

[deae118-B13] Brown HL , Warner JJ, Gianos E, Gulati M, Hill AJ, Hollier LM, Rosen SE, Rosser ML, Wenger NK; American Heart Association and the American College of Obstetricians and Gynecologists. Promoting risk identification and reduction of cardiovascular disease in women through collaboration with obstetricians and gynecologists: a presidential advisory from the American Heart Association and the American College of Obstetricians and Gynecologists. Circulation 2018;137:e843–e852. 29748185 10.1161/CIR.0000000000000582

[deae118-B14] Bubach S , Menezes AM, Barros FC, Wehrmeister FC, Gonçalves H, Assunção MC, Horta BL. Impact of the age at menarche on body composition in adulthood: results from two birth cohort studies. BMC Public Health 2016;16:1007. 27660104 10.1186/s12889-016-3649-x PMC5034580

[deae118-B15] Capers PL , Kinsey AW, Miskell EL, Affuso O. Visual representation of body shape in African-American and European American women: clinical considerations. Clin Med Insights Womens Health 2016;9:63–70. 27478392 10.4137/CMWH.S37587 PMC4955976

[deae118-B16] Cena H , Chiovato L, Nappi RE. Obesity, polycystic ovary syndrome, and infertility: a new avenue for GLP-1 receptor agonists. J Clin Endocrinol Metab 2020;105:e2695–e2709. 32442310 10.1210/clinem/dgaa285 PMC7457958

[deae118-B17] Chen Z , Bassford T, Green SB, Cauley JA, Jackson RD, LaCroix AZ, Leboff M, Stefanick ML, Margolis KL. Postmenopausal hormone therapy and body composition—a substudy of the estrogen plus progestin trial of the Women’s Health Initiative. Am J Clin Nutr 2005;82:651–656. 16155280 10.1093/ajcn.82.3.651

[deae118-B18] Chen Z , Wang Z, Lohman T, Heymsfield SB, Outwater E, Nicholas JS, Bassford T, LaCroix A, Sherrill D, Punyanitya M et al Dual-energy X-ray absorptiometry is a valid tool for assessing skeletal muscle mass in older women. J Nutr 2007;137:2775–2780. 18029498 10.1093/jn/137.12.2775

[deae118-B19] Davis EM , Zyzanski SJ, Olson CM, Stange KC, Horwitz RI. Racial, ethnic, and socioeconomic differences in the incidence of obesity related to childbirth. Am J Public Health 2009;99:294–299. 19059856 10.2105/AJPH.2007.132373 PMC2622775

[deae118-B20] Després JP , Tchernof A. Pathophysiology of human visceral obesity: an update. Physiol Rev 2013;93:359–404. 23303913 10.1152/physrev.00033.2011

[deae118-B21] Dhana K , van Rosmalen J, Vistisen D, Ikram MA, Hofman A, Franco OH, Kavousi M. Trajectories of body mass index before the diagnosis of cardiovascular disease: a latent class trajectory analysis. Eur J Epidemiol 2016;31:583–592. 26955830 10.1007/s10654-016-0131-0 PMC4956703

[deae118-B22] Farland LV , Wang YX, Gaskins AJ, Rich-Edwards JW, Wang S, Magnus MC, Chavarro JE, Rexrode KM, Missmer SA. Infertility and risk of cardiovascular disease: a prospective cohort study. J Am Heart Assoc 2023;12:e027755. 36847044 10.1161/JAHA.122.027755 PMC10111453

[deae118-B23] Fox CS , Massaro JM, Hoffmann U, Pou KM, Maurovich-Horvat P, Liu CY, Vasan RS, Murabito JM, Meigs JB, Cupples LA et al Abdominal visceral and subcutaneous adipose tissue compartments: association with metabolic risk factors in the Framingham Heart Study. Circulation 2007;116:39–48. 17576866 10.1161/CIRCULATIONAHA.106.675355

[deae118-B24] Gepner Y , Shelef I, Schwarzfuchs D, Zelicha H, Tene L, Yaskolka Meir A, Tsaban G, Cohen N, Bril N, Rein M et al Effect of distinct lifestyle interventions on mobilization of fat storage pools: CENTRAL magnetic resonance imaging randomized controlled trial. Circulation 2018;137:1143–1157. 29142011 10.1161/CIRCULATIONAHA.117.030501

[deae118-B25] Gill D , Brewer CF, Del Greco M F, Sivakumaran P, Bowden J, Sheehan NA, Minelli C. Age at menarche and adult body mass index: a Mendelian randomization study. Int J Obes (Lond) 2018;42:1574–1581. 29549348 10.1038/s41366-018-0048-7

[deae118-B26] Grandi SM , Filion KB, Yoon S, Ayele HT, Doyle CM, Hutcheon JA, Smith GN, Gore GC, Ray JG, Nerenberg K et al Cardiovascular disease-related morbidity and mortality in women with a history of pregnancy complications. Circulation 2019;139:1069–1079. 30779636 10.1161/CIRCULATIONAHA.118.036748

[deae118-B27] Grandi SM , Hinkle SN, Mumford SL, Sjaarda LA, Grantz KL, Mendola P, Mills JL, Pollack AZ, Yeung E, Zhang C et al Long-term mortality in women with pregnancy loss and modification by race/ethnicity. Am J Epidemiol 2022;191:787–799. 35136903 10.1093/aje/kwac023 PMC9630116

[deae118-B28] Harville EW , Jacobs M, Shu T, Breckner D, Wallace M. Comparison of reproductive history gathered by interview and by vital records linkage after 40 years of follow-up: Bogalusa babies. BMC Med Res Methodol 2019;19:114. 31164081 10.1186/s12874-019-0758-0 PMC6549375

[deae118-B29] Hays J , Hunt JR, Hubbell FA, Anderson GL, Limacher M, Allen C, Rossouw JE. The Women’s Health Initiative recruitment methods and results. Ann Epidemiol 2003;13:S18–S77. 14575939 10.1016/s1047-2797(03)00042-5

[deae118-B30] He Y , Tian J, Oddy WH, Dwyer T, Venn AJ. Association of childhood obesity with female infertility in adulthood: a 25-year follow-up study. Fertil Steril 2018;110:596–604.e1. 30196944 10.1016/j.fertnstert.2018.05.011

[deae118-B33] Hologic Inc. Visceral Adipose Tissue, Horizon QDR Series Operator Manual Part Number MAN-03644 Revision 005. Mississauga, Ontario, Canada: Hologic, Inc, 2015.

[deae118-B34] Iorga A , Cunningham CM, Moazeni S, Ruffenach G, Umar S, Eghbali M. The protective role of estrogen and estrogen receptors in cardiovascular disease and the controversial use of estrogen therapy. Biol Sex Differ 2017;8:33. 29065927 10.1186/s13293-017-0152-8 PMC5655818

[deae118-B35] Iversen DS , Kesmodel US, Ovesen PG. Associations between parity and maternal BMI in a population-based cohort study. Acta Obstet Gynecol Scand 2018;97:694–700. 29415327 10.1111/aogs.13321

[deae118-B36] Jung AM , Missmer SA, Cramer DW, Ginsburg ES, Terry KL, Vitonis AF, Farland LV. Self-reported infertility diagnoses and treatment history approximately 20 years after fertility treatment initiation. Fertil Res Pract 2021;7:7. 33712085 10.1186/s40738-021-00099-2 PMC7953690

[deae118-B37] Kabat GC , Kim MY, Wactawski-Wende J, Lane D, Wassertheil-Smoller S, Rohan TE. Menstrual and reproductive factors, exogenous hormone use, and risk of thyroid carcinoma in postmenopausal women. Cancer Causes Control 2012;23:2031–2040. 23090034 10.1007/s10552-012-0084-x

[deae118-B38] Lalia AZ , Dasari S, Johnson ML, Robinson MM, Konopka AR, Distelmaier K, Port JD, Glavin MT, Esponda RR, Nair KS et al Predictors of whole-body insulin sensitivity across ages and adiposity in adult humans. J Clin Endocrinol Metab 2016;101:626–634. 26709968 10.1210/jc.2015-2892 PMC4880121

[deae118-B39] Lancet. A broader vision for women’s health. Lancet 2023;402:347. 37516532 10.1016/S0140-6736(23)01570-2

[deae118-B40] Langer RD , White E, Lewis CE, Kotchen JM, Hendrix SL, Trevisan M. The Women’s Health Initiative Observational Study: baseline characteristics of participants and reliability of baseline measures. Ann Epidemiol 2003;13:S107–S121. 14575943 10.1016/s1047-2797(03)00047-4

[deae118-B41] Lau ES , Wang D, Roberts M, Taylor CN, Murugappan G, Shadyab AH, Schnatz PF, Farland LV, Wood MJ, Scott NS et al Infertility and risk of heart failure in the Women’s Health Initiative. J Am Coll Cardiol 2022;79:1594–1603. 35450577 10.1016/j.jacc.2022.02.020 PMC9377329

[deae118-B42] Lee PH. Should we adjust for a confounder if empirical and theoretical criteria yield contradictory results? A simulation study. Sci Rep 2014;4:6085. 25124526 10.1038/srep06085 PMC5381407

[deae118-B43] Liu J , Fox CS, Hickson DA, May WD, Hairston KG, Carr JJ, Taylor HA. Impact of abdominal visceral and subcutaneous adipose tissue on cardiometabolic risk factors: the Jackson Heart Study. J Clin Endocrinol Metab 2010;95:5419–5426. 20843952 10.1210/jc.2010-1378 PMC2999970

[deae118-B44] Ma W , Zhu H, Yu X, Zhai X, Li S, Huang N, Liu K, Shirai K, Sheerah HA, Cao J. Association between android fat mass, gynoid fat mass and cardiovascular and all-cause mortality in adults: NHANES 2003-2007. Front Cardiovasc Med 2023;10:1055223. 37273879 10.3389/fcvm.2023.1055223 PMC10233278

[deae118-B45] Mattick LJ , Bea JW, Singh L, Hovey KM, Banack HR, Wactawski-Wende J, Manson JE, Funk JL, Ochs-Balcom HM. Serum follicle-stimulating hormone and 5-year change in adiposity in healthy postmenopausal women. J Clin Endocrinol Metab 2022;107:e3455–e3462. 35435955 10.1210/clinem/dgac238 PMC9282244

[deae118-B0502014] Mishra GD, , Cooper R, , Kuh D. A life course approach to reproductive health: theory and methods. Maturitas 2010;65:92–97. 20079587 10.1016/j.maturitas.2009.12.009 PMC3504662

[deae118-B47] Mishra GD , Cooper R, Tom SE, Kuh D. Early life circumstances and their impact on menarche and menopause. Womens Health (Lond) 2009;5:175–190. 19245355 10.2217/17455057.5.2.175 PMC3287288

[deae118-B48] Mishra SR , Chung H-F, Waller M, Mishra GD. Duration of estrogen exposure during reproductive years, age at menarche and age at menopause, and risk of cardiovascular disease events, all-cause and cardiovascular mortality: a systematic review and meta-analysis. BJOG 2021;128:809–821. 32965759 10.1111/1471-0528.16524

[deae118-B49] Murphy N , Xu L, Zervoudakis A, Xue X, Kabat G, Rohan TE, Wassertheil-Smoller S, O’Sullivan MJ, Thomson C, Messina C et al Reproductive and menstrual factors and colorectal cancer incidence in the Women’s Health Initiative Observational Study. Br J Cancer 2017;116:117–125. 27898658 10.1038/bjc.2016.345 PMC5220139

[deae118-B50] Murugappan G , Leonard SA, Farland LV, Lau ES, Shadyab AH, Wild RA, Schnatz P, Carmichael SL, Stefanick ML, Parikh NI. Association of infertility with atherosclerotic cardiovascular disease among postmenopausal participants in the Women’s Health Initiative. Fertil Steril 2022;117:1038–1046. 35305814 10.1016/j.fertnstert.2022.02.005 PMC9081220

[deae118-B51] Pacyga DC , Henning M, Chiang C, Smith RL, Flaws JA, Strakovsky RS. Associations of pregnancy history with BMI and weight gain in 45-54-year-old women. Curr Dev Nutr 2019;4:nzz139. 31893261 10.1093/cdn/nzz139 PMC6933615

[deae118-B52] Parikh NI , Gonzalez JM, Anderson CAM, Judd SE, Rexrode KM, Hlatky MA, Gunderson EP, Stuart JJ, Vaidya D; American Heart Association Council on Epidemiology and Prevention; Council on Arteriosclerosis, Thrombosis and Vascular Biology; Council on Cardiovascular and Stroke Nursing; and the Stroke Council. Adverse pregnancy outcomes and cardiovascular disease risk: unique opportunities for cardiovascular disease prevention in women: a scientific statement from the American Heart Association. Circulation 2021;143:e902–e916. 33779213 10.1161/CIR.0000000000000961

[deae118-B53] Peters SA , Huxley RR, Woodward M. Women’s reproductive health factors and body adiposity: findings from the UK Biobank. Int J Obes (Lond) 2016;40:803–808. 26700411 10.1038/ijo.2015.254

[deae118-B54] Pierce MB , Leon DA. Age at menarche and adult BMI in the Aberdeen children of the 1950s cohort study. Am J Clin Nutr 2005;82:733–739. 16210700 10.1093/ajcn/82.4.733

[deae118-B55] Porter SA , Massaro JM, Hoffmann U, Vasan RS, O’Donnel CJ, Fox CS. Abdominal subcutaneous adipose tissue: a protective fat depot? Diabetes Care 2009;32:1068–1075. 19244087 10.2337/dc08-2280 PMC2681034

[deae118-B56] Pradhan AD. Sex differences in the metabolic syndrome: implications for cardiovascular health in women. Clin Chem 2014;60:44–52. 24255079 10.1373/clinchem.2013.202549

[deae118-B57] Reagan PB , Salsberry PJ, Fang MZ, Gardner WP, Pajer K. African-American/white differences in the age of menarche: accounting for the difference. Soc Sci Med 2012;75:1263–1270. 22726619 10.1016/j.socscimed.2012.05.018 PMC3407312

[deae118-B58] Rich-Edwards JW. Reproductive health as a sentinel of chronic disease in women. Womens Health (Lond) 2009;5:101–105. 19245346 10.2217/17455057.5.2.101

[deae118-B59] Sam AH , Dhillo WS. Endocrine links between fat and reproduction. Obstet Gynaecol 2010;12:231–236.

[deae118-B60] Shah RV , Murthy VL, Abbasi SA, Blankstein R, Kwong RY, Goldfine AB, Jerosch-Herold M, Lima JA, Ding J, Allison MA. Visceral adiposity and the risk of metabolic syndrome across body mass index: the MESA study. JACC Cardiovasc Imaging 2014;7:1221–1235. 25440591 10.1016/j.jcmg.2014.07.017 PMC4268163

[deae118-B90993953] Stefanick ML, , Cochrane BB, , Hsia J, , Barad DH, , Liu JH, , Johnson SR. The Women’s Health Initiative postmenopausal hormone trials: overview and baseline characteristics of participants. Ann Epidemiol 2003;13:S78–S86. 14575940 10.1016/s1047-2797(03)00045-0

[deae118-B61] Tchkonia T , Giorgadze N, Pirtskhalava T, Tchoukalova Y, Karagiannides I, Forse RA, DePonte M, Stevenson M, Guo W, Han J et al Fat depot origin affects adipogenesis in primary cultured and cloned human preadipocytes. Am J Physiol Regul Integr Comp Physiol 2002;282:R1286–R1926. 11959668 10.1152/ajpregu.00653.2001

[deae118-B62] Trikudanathan S , Pedley A, Massaro JM, Hoffmann U, Seely EW, Murabito JM, Fox CS. Association of female reproductive factors with body composition: the Framingham Heart Study. J Clin Endocrinol Metab 2013;98:236–244. 23093491 10.1210/jc.2012-1785 PMC3537091

[deae118-B63] VanderWeele TJ. Principles of confounder selection. Eur J Epidemiol 2019;34:211–219. 30840181 10.1007/s10654-019-00494-6 PMC6447501

[deae118-B64] Wilding JPH , Batterham RL, Calanna S, Van Gaal LF, McGowan BM, Rosenstock J, Tran MTD, Wharton S, Yokote K, Zeuthen N et al Impact of semaglutide on body composition in adults with overweight or obesity: exploratory analysis of the STEP 1 study. J Endocr Soc 2021;5(Suppl 1):A16–A17.

[deae118-B65] Xu F , Earp JE, Adami A, Blissmer BJ, Riebe D, Greene GW. Sex and race/ethnicity specific reference predictive equations for abdominal adiposity indices using anthropometry in US adults. Nutr Metab Cardiovasc Dis 2023;33:956–966. 36958968 10.1016/j.numecd.2023.03.001

[deae118-B66] Zajacova A , Ailshire J. Body mass trajectories and mortality among older adults: a joint growth mixture-discrete-time survival analysis. Gerontologist 2014;54:221–231. 23355450 10.1093/geront/gns164 PMC3954412

