# Supplementary material for: The association between reproductive history and abdominal adipose tissue among postmenopausal women: results from the Women’s Health Initiative
Source: Hum Reprod. 2024 Jun 18;39(8):1804–15. doi: 10.1093/humrep/deae118 (PMC11291955; doi:10.1093/humrep/deae118)
Supplement: deae118_Supplementary_Figure_S1 [file deae118_supplementary_figure_s1.pdf]

Human Reproduction, 2024, 1–1
https://doi.org/10.1093/humrep/deae118
Supplementary Data

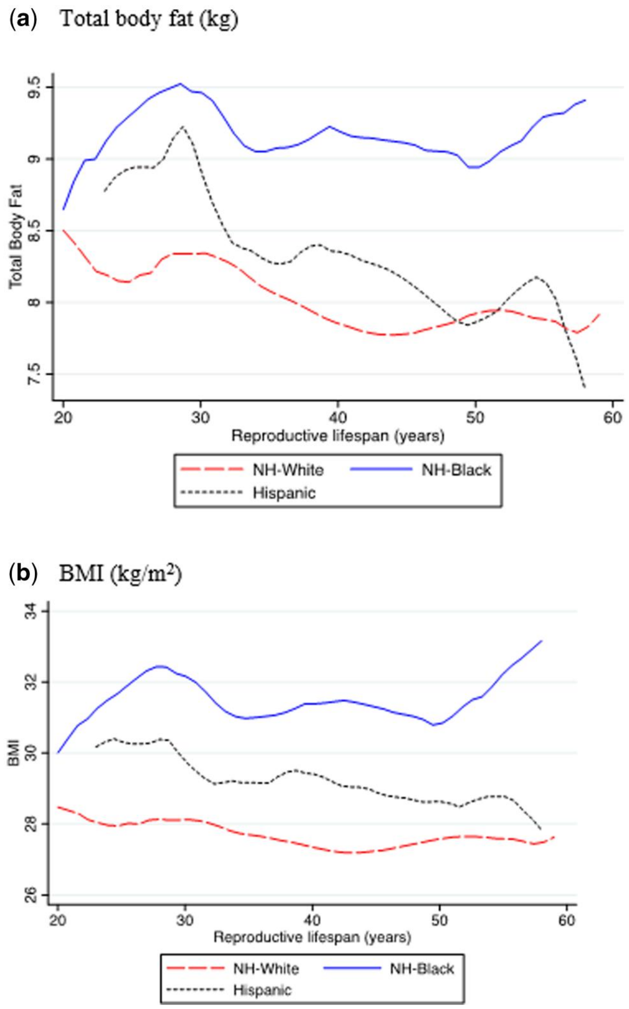

Supplementary Figure S1. A comparison of total body fat, BMI, and reproductive lifespan according to race and ethnicity. NH, non-Hispanic.
