# Supplementary material for: The association between reproductive history and abdominal adipose tissue among postmenopausal women: results from the Women’s Health Initiative
Source: Hum Reprod. 2024 Jun 18;39(8):1804–15. doi: 10.1093/humrep/deae118 (PMC11291955; doi:10.1093/humrep/deae118)
Supplement: deae118_Supplementary_Table_S1 [file deae118_supplementary_table_s1.pdf]

OXFORD
human reproduction
Human Reproduction, 2024, 1–1
https://doi.org/10.1093/humrep/deae118
Supplementary Data

Supplementary Table S1. Baseline demographic and anthropometric characteristics of the study sample overall and stratified by race-ethnicity.

	Overall	Non-Hispanic White	Non-Hispanic Black	Hispanic/Latina
**N**	11, 020	8369	1492	715
**Age** (mean, SD, years)	62.4 (7.4)	63.9 (7.3)	62.0 (7.4)	60.7 (4.0)
**Completed high school or equivalent** (%)	68.5	71.5	62.0	48.0
**Household income** (%)				
<$35 000	17.1	9.0	42.7	46.3
$35 000–75 000	57.0	62.9	40.4	33.9
>$75 000	25.9	28.0	16.9	19.8
**Marital status** (%)				
Married or with partner	61.7	64.9	43.7	61.9
**Age at menopause** (mean, SD, years)	47.5 (6.9)	47.8 (6.7)	45.9 (7.5)	47.7 (7.5)
**Height** (mean, SD, cm)	161.6 (6.4)	161.7 (6.3)	162.7 (5.9)	157.9 (5.7)
**Hormone therapy use** (%)				
Never	47.8	45.0	57.7	55.5
Former	15.9	16.5	14.4	11.6
Current	36.4	38.5	27.9	32.8
**Smoking Status** (%)				
Never	54.7	53.4	56.9	64.0
Former	37.3	39.0	33.0	28.4
Current	8.0	7.6	10.1	7.6
**Alcohol intake** (mean, SD, servings per week)	1.74 (4.1)	2.0 (4.3)	0.8 (3.2)	1.0 (3.1)
**Recreational physical activity** (mean, SD, MET-hours/week)	11.5 (13.9)	12.4 (14.2)	7.7 (11.4)	9.6 (12.4)
**Physical function**	78.2 (21.4)	79.9 (20.1)	69.8 (25.3)	77.8 (22.0)
**Waist circumference** (mean, SD, cm)	85.8 (13.4)	84.7 (2.7)	91.7 (13.0)	86.4 (12.5)
**BMI** (mean, SD, kg/m^2)	28.2 (5.9)	27.6 (5.6)	31.4 (6.4)	29.1 (5.8)
BMI category (%, kg/m^2)				
<18.5	0.8	0.9	0.5	0.7
18.5–24.9	32.0	35.8	15.0	23.5
25–29.9	35.1	35.7	30.8	38.5
>30–34.9	19.8	17.9	28.0	23.3
35–39.9	8.1	6.7	15.0	9.4
>40	4.2	3.0	10.7	4.6

MET, metabolic equivalent task.
