# Supplementary material for: The association between reproductive history and abdominal adipose tissue among postmenopausal women: results from the Women’s Health Initiative
Source: Hum Reprod. 2024 Jun 18;39(8):1804–15. doi: 10.1093/humrep/deae118 (PMC11291955; doi:10.1093/humrep/deae118)
Supplement: deae118_Supplementary_Table_S2 [file deae118_supplementary_table_s2.pdf]

OXFORD
human reproduction

Human Reproduction, 2024, 1–1
https://doi.org/10.1093/humrep/deae118
Supplementary Data

Supplementary Table S2. Participant characteristics according to age of menarche.

	≤9 years	10 years	11 years	12 years	13 years	14 years	≥ 15 years
**Age** (median, Q1–Q3)	58 (54–58)	61 (56–61)	62 (56–62)	63 (57–63)	64 (57–64)	64 (58–64)	65 (58–65)
**Race** (%)							
American Indian/ Alaskan Native/Native Hawaiian/Other	1.7	1.2	1.4	1.1	0.7	0.9	1.0
Asian	0.0	0.5	0.1	0.2	0.4	0.4	0.5
Black	25.2	13.7	13.8	13.2	11.8	14.6	18.2
More than one race	0.8	0.9	0.7	0.8	1.0	0.80	0.6
White	72.3	83.8	84.0	84.8	86.1	83.3	79.8
**Ethnicity** (%)							
Hispanic/Latino	10.9	5.9	7.2	6.8	6.2	7.3	6.7
**Education** (%)							
High school diploma	27.3	29.6	28.4	28.6	30.8	32.5	37.7
Some college	35.2	36.3	37.9	37.9	36.8	38.1	38.3
College graduate or higher	35.9	33.9	33.2	32.9	31.8	28.7	22.9
Unknown	1.6	0.2	0.5	0.6	0.6	0.7	1.1
**Smoking ever** (%)	50.0	45.9	44.7	45.6	45.5	45.8	46.6
**Alcohol** (g/day)	0.035 (0.007–0.035)	0.069 (0.01–0.069)	0.14 (0.012–0.14)	0.15 (0.012–0.15)	0.42 (0.013–0.42)	0.15 (0.012–0.15)	0.09 (0.011–0.09)
**Physical activity** (kcal/week per kg)	5 (0.75–5)	8.2 (1.5–8.2)	7.3 (1.5–7.3)	7 (1.5–7)	7.5 (1.9–7.5)	6.8 (1.2–6.8)	7 (1.5–7)
**Diet quality** (Total HEi 2015 score)	63.3 (55.1–63.3)	63.5 (56.2–63.5)	64.3 (56.4–64.3)	63.7 (55.8–63.7)	64.9 (56.8–64.9)	63.7 (55.7–63.7)	62.2 (54.5–62.2)
**Diabetes** (%)	15.6	7.6	9.3	8.2	5.8	6.3	8.2
**Cardiovascular disease** (%)	25.7	20.6	18.1	19.4	18.5	18.7	21.5
**Cancer** (%)	15.1	5.6	9.0	7.1	6.1	6.8	7.5

Q1–Q3, quartile 1–quartile 3; HEI, healthy eating index.
