# Supplementary material for: The association between reproductive history and abdominal adipose tissue among postmenopausal women: results from the Women’s Health Initiative
Source: Hum Reprod. 2024 Jun 18;39(8):1804–15. doi: 10.1093/humrep/deae118 (PMC11291955; doi:10.1093/humrep/deae118)
Supplement: deae118_Supplementary_Table_S3 [file deae118_supplementary_table_s3.pdf]

OXFORD

human reproduction

Human Reproduction, 2024, 1–1
https://doi.org/10.1093/humrep/deae118
Supplementary Data

Supplementary Table S3. Participant characteristics according to parity.

	Never pregnant	0–No term	1	2	3	4	5+
**Age** (median, Q1–Q3)	65 (57–65)	62 (54–62)	62 (55–62)	62 (55–62)	63 (57–63)	64 (58–64)	64 (59–64)
**Race** (%)							
American Indian/ Alaskan Native/Native Hawaiian/Other	0.8	0.5	0.9	0.5	0.6	1.2	2.3
Asian	0.4	0.9	0.2	0.5	0.3	0.2	0.1
Black	10.6	28.3	21.2	11.1	10.1	11.5	21.0
More than one race	0.5	2.3	1.2	1.0	0.8	0.5	0.7
White	87.7	68.0	76.5	86.9	88.2	86.6	75.9
**Ethnicity** (%)							
Hispanic/Latino	6.1	7.2	5.3	3.7	5.5	7.2	13.1
**Education** (%)							
High school diploma	18.7	22.0	25.0	26.0	29.1	34.5	47.0
Some college	32.9	37.2	39.6	38.8	38.2	38.4	35.7
College graduate or higher	48.0	39.5	34.6	34.6	32.1	26.4	16.7
Unknown	0.5	1.3	0.8	0.5	0.6	0.7	0.6
**Smoking ever** (%)	45.9	57.0	49.6	48.1	46.0	42.7	40.7
**Alcohol** (g/day)	0.84 (0.016–0.84)	0.1 (0.01–0.1)	0.11 (0.011–0.11)	0.15 (0.013–0.15)	0.44 (0.014–0.44)	0.12 (0.013–0.12)	0.056 (0.0099–0.056)
**Physical activity** (kcal/week per kg)	8 (1.9–8)	5 (0.94–5)	7 (1.2–7)	7.5 (2.2–7.5)	7.5 (1.9–7.5)	8.2 (1.9–8.2)	5.8 (0.75–5.8)
**Diet quality** (Total HEi 2015 score)	65 (58–65)	64 (56–64)	64 (56–64)	65 (56–65)	64 (57–64)	64 (56–64)	62 (54–62)
**Diabetes** (%)	7.8	8.1	6.3	6.3	5.7	7.3	12.3
**Cardiovascular disease** (%)	19.2	17.0	17.4	18.5	19.5	20.1	20.8
**Cancer** (%)	9.1	7.3	9.0	7.5	5.9	8.1	5.6

Q1–Q3, quartile 1–quartile 3; HEI, healthy eating index.

© The Author(s) 2024. Published by Oxford University Press on behalf of European Society of Human Reproduction and Embryology.
This is an Open Access article distributed under the terms of the Creative Commons Attribution License (https://creativecommons.org/licenses/by/4.0/), which permits unrestricted reuse, distribution, and reproduction in any medium, provided the original work is properly cited.
Human Reproduction, 2024, 00, 1–12
https://doi.org/10.1093/humrep/deae118
Original Article
